# Mutations in *NOTCH3* Gene may Promote the Clinical Presentation of Spinocerebellar Ataxia Type 37 Caused by Mutations in *DAB1* Gene

**DOI:** 10.3389/fmolb.2021.668312

**Published:** 2021-06-16

**Authors:** Zhao-Wei Wang, Li-Ping Wang, Ye Du, Qi Liu

**Affiliations:** ^1^ Department of Neurology, Shaoxing People’s Hospital (Shaoxing Hospital, Zhejiang University School of Medicine), Shaoxing, China; ^2^ Department of Transfusion, Shaoxing People’s Hospital (Shaoxing Hospital, Zhejiang University School of Medicine), Shaoxing, China

**Keywords:** SAC37, CADASIL, Dab1, Notch3, rare diseases

## Abstract

**Background:** Autosomal dominant spinocerebellar ataxia type 37 (SCA37) and Cerebral autosomal dominant arteriopathy with subcortical infarct and leukoencephalopathy (CADASIL) result from *DAB1* and *NOTCH3* gene mutations, respectively.

**Methods:** In addition to conventional diagnostic methods, next-generation sequencing (NGS) and Sanger sequencing were performed to define and confirm the *DAB1* and *NOTCH3* gene mutation for a Chinese pedigree. Bioinformatics analysis was also applied for the mutated DAB1 and NOTCH3 protein using available software tools.

**Results:** Brain magnetic resonance imaging shows diffuse leukoencephalopathy and cerebellar atrophy in the proband. NGS and Sanger sequencing identified two novel heterozygous mutations: NM_021080:c.318T > G (*p*.H106Q) in the *DAB1* gene and NM_000435:c.3298C > T (*p*.R1100C) in the *NOTCH3* gene. Bioinformatics analysis suggested that the *DAB1* and *NOTCH3* gene mutations are disease-causing and may be responsible for the phenotypes.

**Conclusion:** This is the first report of a pedigree with both SAC37 and CADASIL phenotypes carrying corresponding gene mutations. Mutations in the *NOTCH3* gene may promote the clinical presentation of spinocerebellar ataxia type 37 caused by mutations in the *DAB1* gene. In addition to general examinations, it is vital for physicians to apply molecular genetics to get an accurate diagnosis in the clinic, especially for rare diseases.

## Introduction

Autosomal dominant spinocerebellar ataxia type 37 (SCA37) is characterized by adult-onset dysarthria, slow progressive gait, limb ataxia with severe dysmetria in the lower extremities, mild dysmetria in the upper extremities, dysphagia, and abnormal ocular movements (Matilla-Duenas et al., 1993; Corral-Juan et al., 2018). The phenotypic manifestations of SCA37 are not specific and no formal diagnostic criteria exist. *DAB1* gene mutations were proposed as the underlying genetic cause in the SCA37 (Corral-Juan et al., 2018; Loureiro et al., 2019).

Cerebral autosomal dominant arteriopathy with subcortical infarct and leukoencephalopathy (CADASIL) is an autosomal dominant inherited vasculopathy characterized by migraine with aura, recurrent subcortical ischemic stroke, cognitive decline, and psychiatric disorders (Chen et al., 2019; Dorszewska et al., 2020). CADASIL is caused by mutations in the *NOTCH3* gene which is localized on chromosome 19p13 (Joutel et al., 1997; Sari et al., 2019).

Here we reported a pedigree that showed both phenotypes of SCA37 and CADASIL. The diagnosis of SCA37 or CADASIL is dependent on molecular genetic testing and radiographic studies.

## Materials and Methods

### Patient and Characteristics

The proband was a 49-year-old male. He was hospitalized because of dizziness accompanied by unstable walking and abnormal vocalization. Two years ago, he gradually developed dizziness when walking, a slow pace, fear of falling, and abnormal voice. However, the patient did not cough due to drinking water, nor did he suffer from weakness and numbness. He had had hypertension for more than 3 years. He had also been a smoker for more than 20 years. The index finger and ring finger of the right hand were missing due to trauma. Physical examination revealed that he was conscious, his temperature was 36.8°C, and his blood pressure was 135/105 mmHg. His neck resistance sign was negative. The pupils on both sides were 3 mm, and are sensitive to both direct and indirect light reflection. His pronunciation was not clear, but the throat reflex was normal. The muscle tone of his limbs was normal, the muscle strength of both upper limbs was level 5, and the muscle strength of both lower limbs was level 4. Both knee tendon reflexes were hyperactive. The bilateral Babinski sign was positive. Pain, touch, and position of the limbs was normal. The bilateral heel knee shin test was abnormal. Doctors were unable to complete a Romberg sign examination with eyes open.

The proband received a thorough examination in our hospital, including head magnetic resonance imaging (MRI), electroencephalogram (EEG), electromyography (EMG), and echocardiography (ECHO).

### Sample Collection

The subject’s consent for the study was obtained according to the Declaration of Helsinki and was approved by the ethical committee of the Shaoxing People’s Hospital. People with normal physical examination acted as controls.

Genotyping DNA was extracted from fasting blood samples using the genomic DNA kit (TIANGEN BIOTECH, Beijing, China, DP304), according to the manufacturer’s instructions.

### Genetic Analysis

The specimen was submitted to Oumeng V Medical Laboratory and whole-exome sequencing (WES) was performed. The WES was performed by Agilent SureSelect Human All Exon V6 kits and Illumina NovaSeq 6000 sequencing platform. The paired-end reads (PE150) were aligned to a Genome Reference Consortium Human Genome Build 37 (GRCh37)-derived alignment set including decoy sequences using the Burrows-Wheeler Aligner (BWA). Single nucleotide variants (SNV’s), small insertions and deletions (indels), and copy number variants were called with GATK Best Practices. The sequencing data for all samples underwent standard quality control checksAn average coverage depth more than 100X, 90.00% of the target region sequencing depth >20X, and Q30 not <90% must be achieved. The pathogenicity of the variants was estimated using the American College of Medical Genetics and Genomics (ACMG) guidelines. Sanger sequencing was used to validate the mutations in the *DAB1* and *NOTCH3* of the patient’s family members and 30 people with normal physical examination in our hospital.

### Bioinformatics Analysis

Bioinformatics analysis was also performed for the mutated DAB1 and NOTCH3 protein using available software tools. The pathogenicity prediction of *DAB1* and *NOTCH3* gene mutations was performed by online bioinformatics software SIFT, REVEL, Polyphen-2, LR pred, Mutation Taster, and Ljb23_metasvm. Clustal X1.83 software was used to analyze evolution conservation among different paralogs and orthologs. Sequences were obtained from https://www.ncbi.nlm.nih.gov/. An iterative threading assembly refinement (I-TASSER) server was used to predict the structure and function of mutated and normal protein. The protein structure was predicted by using SWISS-MODEL (https://swissmodel.expasy.org/) online program. The STRING database (version 11.0) was used to predict protein-protein interactions of DAB1 and NOTCH3 proteins; the minimum required interaction score and number were set to 0.700 and 10, respectively.

## Results

**FIGURE 1:**
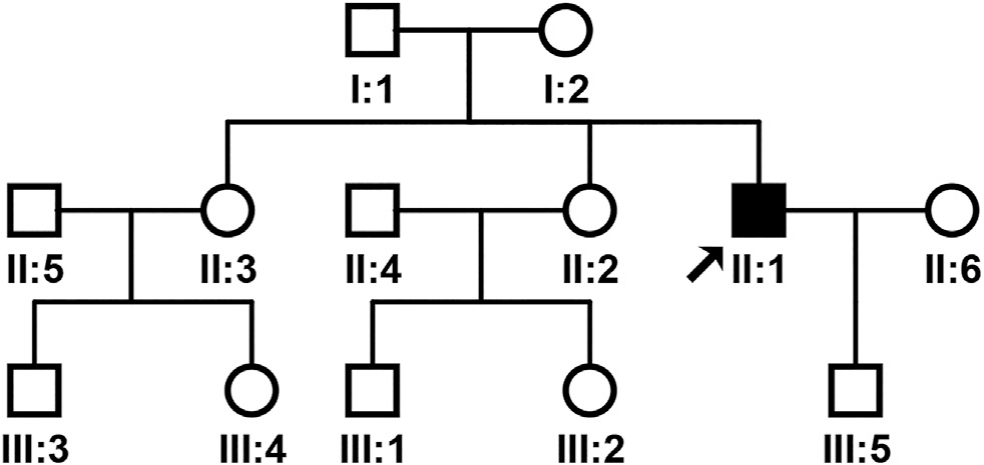
Pedigree of the patient’s family. The arrow indicates the proband.

**FIGURE 2:**
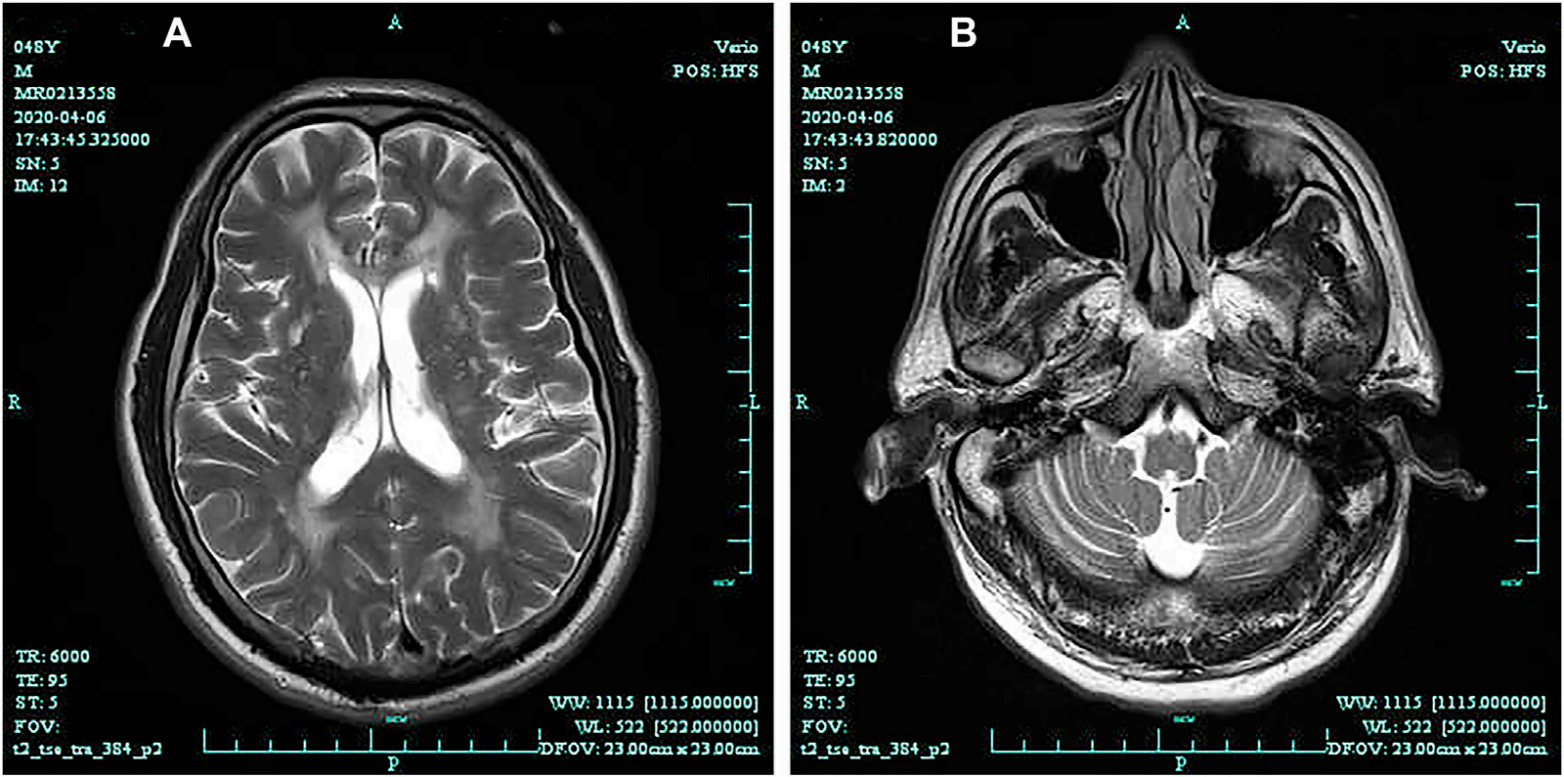
Brain magnetic resonance imaging (T2-weighted) shows diffuse leukoencephalopathy **(A)**; Brain magnetic resonance (T2-weighted) shows cerebellar atrophy **(B)**.

The pedigree of the family was shown in Figure 1. The proband’s brain MRI was shown in Figure 2.

**FIGURE 3:**
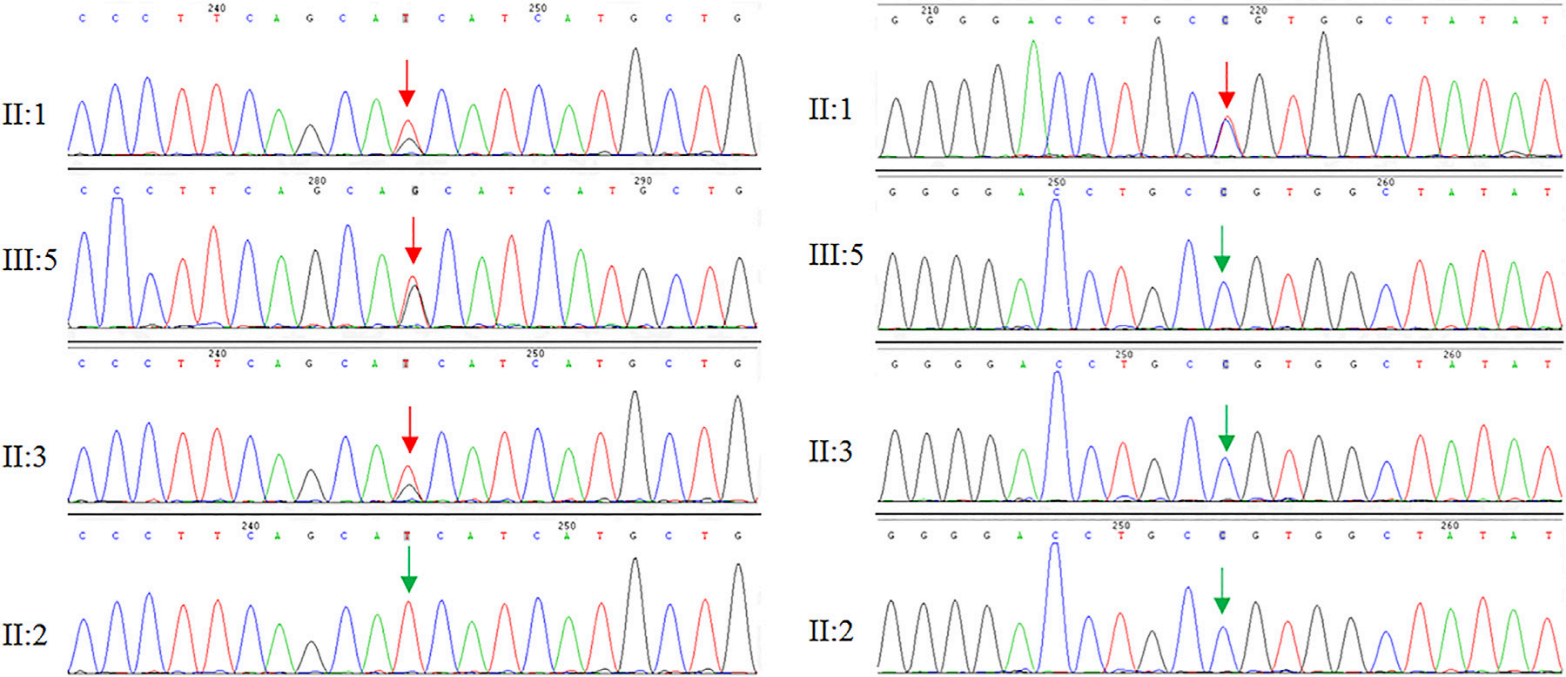
Genome sequencing revealed two heterozygous mutations in the DAB1 and NOTCH3 gene, respectively.

**FIGURE 4:**
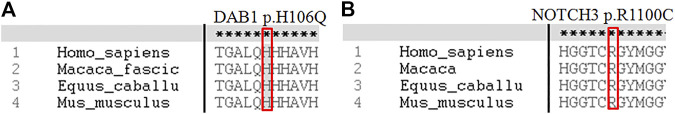
Evolutionary conservation analysis of multiple sequence alignments of DAB1 **(A)** and NOTCH3 **(B)** protein. The comparison between different eukaryotic species, showing that the base pair involving the nucleotide at position 318 in DAB1 and 3298 in NOTCH3 are highly conserved.

WES and Sanger sequencing identified that the proband carried two novel heterozygous mutations: NM_021080:c.318T > G (*p*.H106Q) in the *DAB1* gene and NM_000435:c.3298C > T (*p*.R1100C) in the *NOTCH3* gene. The proband’s son and one sister carried NM_021080:c.318T > G (*p*.H106Q) in the *DAB1* gene, while no *DAB1* and *NOTCH3* gene mutations were detected in family members and healthy control people (Figure 3). The predicted results for *NOTCH3* gene mutation using online bioinformatics software, including SIFT, REVEL, Polyphen-2, LR pred, Mutation Taster, and Ljb23_metasvm, were 0.019, 0.616, 0.846, 0.763, 1, and 0.484, respectively. All the results suggested that the *NOTCH3* gene mutation is disease-causing and may be responsible for the phenotypes. Evolutionary conservation analysis of multiple sequence alignments of DAB1 and NOTCH3 protein and their homologs showed that the amino acid at residue 106 and 1,100 was evolutionarily conserved from monkey to human (Figure 4), suggesting that amino acids may play an important role in the function of the DAB1 and NOTCH3 protein.

**FIGURE 5:**
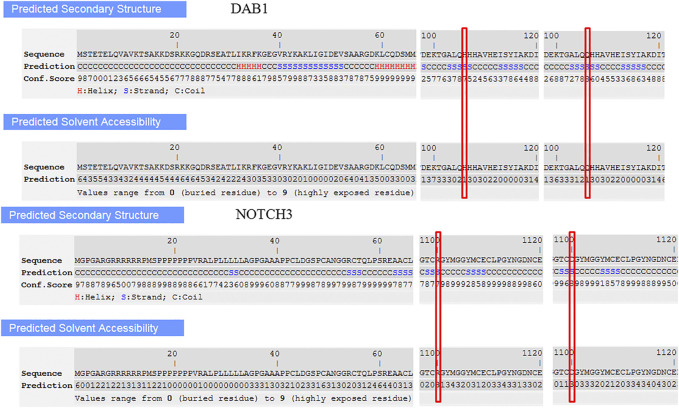
Secondary structure and solvent accessibility of wild and mutant type in DAB1 and NOTCH3 gene predicted by I-TASSER server, respectively.

**FIGURE 6:**
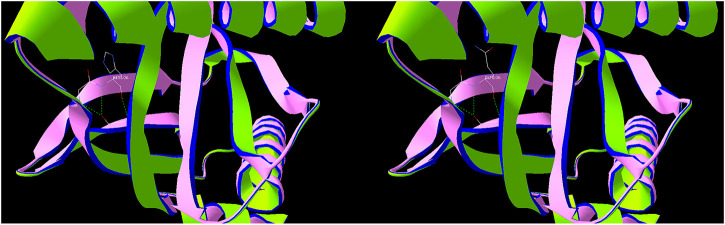
Predicted protein structure of DAB1 using the SWISS-MODEL server. Green indicates hydrogen bonds, used to connect amino acids.

Secondary structure was predicted to be strand by I-TASSER server with a high confidence score for this variant. Solvent accessibility predicted that both normal and mutant amino acids at this position are buried in protein; the accessibility to solvent of each of these amino acids is 4 (Figure 5). SWISS-MODEL workspace demonstrated that the alterations resulting from the mutations in the *DAB1* gene altered the protein structure (Figure 6), however, SWISS-MODEL can not model missing terminal residues because there are no residues (coordinates) available from the template of the *NOTCH3* gene.

**FIGURE 7:**
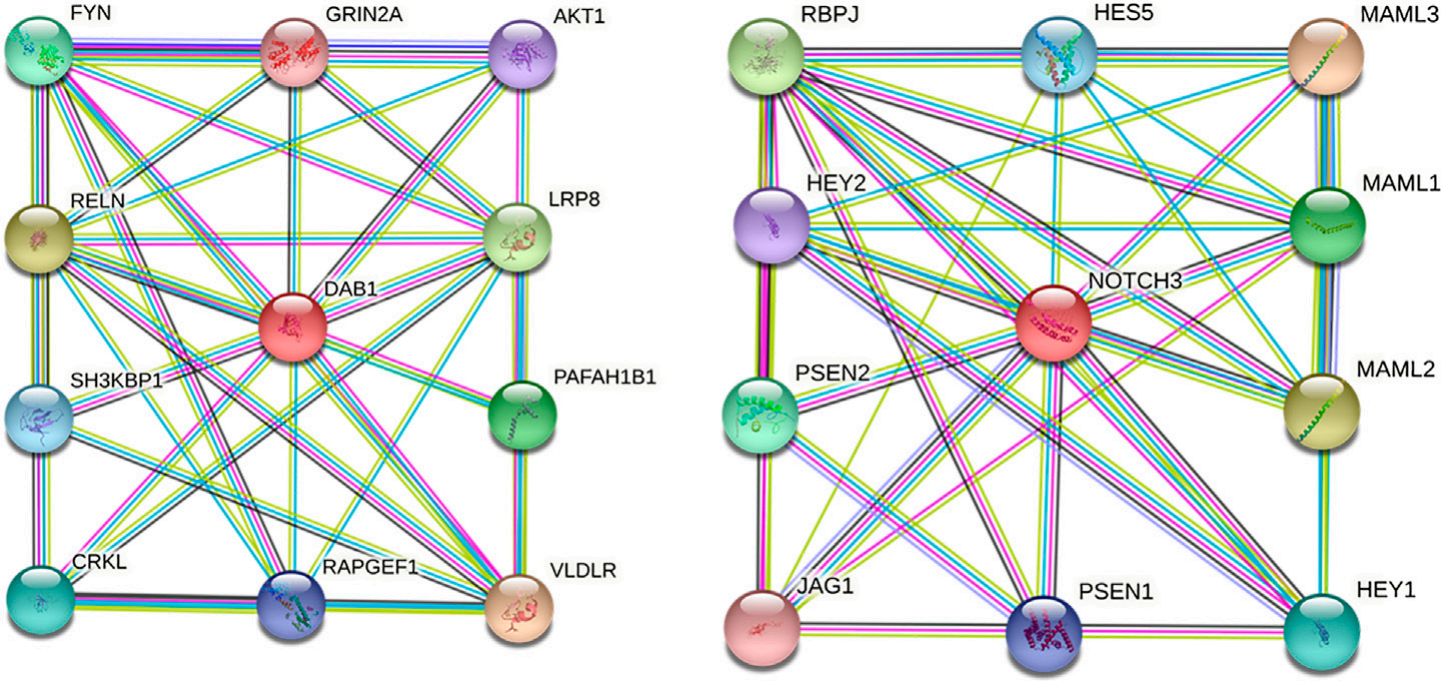
Protein–protein interaction network of DAB1 and NOTCH3. Predicted functional partners are as follows: VLDLR, very low-density lipoprotein receptor, binds VLDL and transports it into cells by endocytosis; RELN, Reelin, extracellular matrix serine protease that plays a role in layering of neurons in the cerebral cortex and cerebellum; LRP8, low-density lipoprotein receptor-related protein 8; PAFAH1B1, Platelet-activating factor acetylhydrolase IB subunit alpha; FYN, tyrosine-protein kinase Fyn, non-receptor tyrosine-protein kinase that plays a role in many biological processes; CRKL, Crk-like protein; SH3KBP1, SH3 domain-containing kinase-binding protein 1, adapter protein involved in regulating diverse signal transduction pathways; RAPGEF1, Rap guanine nucleotide exchange factor 1, guanine nucleotide-releasing protein; AKT1, RAC-alpha serine/threonine-protein kinase; GRIN2A, Glutamate receptor ionotropic, NMDA 2A, component of NMDA receptor complexes; MAML3, Mastermind-like protein 3, acts as a transcriptional coactivator for NOTCH proteins; MAML2, Mastermind-like protein 2, acts as a transcriptional coactivator for NOTCH proteins; RBPJ, recombining binding protein suppressor of hairless; MAML1, Mastermind-like protein 1, acts as a transcriptional coactivator for NOTCH proteins; PSEN2, Presenilin-2, probable catalytic subunit of the gamma-secretase complex; HEY1, hairy/enhancer-of-split related with YRPW motif protein 1; HESS, transcription factor HES-5; PSEN1, Presenilin-1, catalytic subunit of the gamma-secretase complex; HEY2, hairy/enhancer-of-split related with YRPW motif protein 2; JAG1, Protein jagged-1, ligand for multiple Notch receptors and involved in the mediation of Notch signaling.

Moreover, interactions among DAB1 or NOTCH3 and respective proteins using the STRING database suggested the potential effects on the development of cardiomyopathies. Ten functional partners were predicted to interact with DAB1 and NOTCH3, as shown in Figure 7.

## Discussion

To date, 66 affected individuals and seven asymptomatic individuals with the ATTTC repeat insertion within DAB1 have been reported from the south of the Iberian Peninsula, and no cases with SCA37 from other areas have been reported (Seixas et al., 2017; Corral-Juan et al., 2018). The estimated prevalence of SCA37 has been reported as 0.20/100,000 in Portugal (Matilla-Duenas et al., 1993; Coutinho et al., 2013). The incidence and prevalence of CADASIL are difficult to investigate and the reported data are limited since CADASIL is clinically and genetically heterogeneous (Kilarski et al., 2015; Rutten et al., 2016; Tang et al., 2019). To date, more than 300 mutations of *NOTCH3* have been recorded in CADASIL patients (Hung et al., 2018).

The mechanism of SCA37 caused by ATTTC insertion in DAB1 showed that the insertion creates new putative XBP1 transcription factor binding motifs, causes RNA-switch and overexpression of specific RNA isoforms in the SCA37 cerebellar cortex, and alters PI3K/AKT signaling (Sánchez et al., 2016; Corral-Juan et al., 2018). The main pathogenic mechanism of CADASIL is the NOTCH3 extracellular domain aggregation (Ma et al., 2020).

In our study, the proband’s parents did not have the phenotypic manifestations of SCA37, different from previously reported individuals diagnosed with SCA37 that have an affected parent. The proband, his one sister, and son have heterozygous mutation c.318T > G (*p*.H106Q) in the *DAB1* gene, while another sister did not have the mutation. Genetic testing only showed the proband carrying heterozygous mutation c.3298C > T (*p*.R1100C) in the *NOTCH3* gene. The pedigree had no phenotypic manifestations of either SCA37 or CADASIL, although the proband had both phenotypes, suggesting *NOTCH3* gene mutations not only cause CADASIL, but also play an important role in the progression of SCA37.

The NM_000435:c.3298C > T heterozygous mutation of the NOTCH3 gene occurs in exon 20, making the arginine at position 1,100 mutated to cysteine, which is located in the EGF-like domain. This can lead to an odd number of cysteine residues in the domain. It has been reported that each EGF-like domain contains six highly conserved cysteine residues. Generally, only the variation in this domain can cause the pathogenesis of CADASIL, and most of them are due to the odd number of cysteine residues, which changes the protein conformation and interferes with signal transmission, resulting in the development and dysfunction of vascular smooth muscle (PM1). This mutation has not been included in the ExAC database, Esp6500 database, 1,000 Genomes Project database, and other population databases, and was not a polymorphic locus (PM2). This variant is not included in disease databases such as ClinVar. Bioinformatics analysis predicts that the variant is pathogenic (PP3). Based on the above evidence, according to the ACMG sequence mutation interpretation guidelines PM1 + PM2 + PP3, it is recommended that the mutation be identified as unclear clinical significance.

The clinical manifestation of the proband was dizziness with walking instability for two years, which is consistent with the CADASIL. However, as this clinical manifestation may also occur in other diseases, more laboratory tests are recommended so the possibility of other diseases can be ruled out.

For autosomal dominant diseases, a single pathogenic mutation can lead to the occurrence of the disease. The NM_000435:c.3298C > T heterozygous mutation of the NOTCH3 gene detected in this study had an unclear clinical significance. It is suggested that family history, clinical phenotype, and genetic characteristics of family members should be collected for family verification, to provide more evidence for mutation site interpretation and disease diagnosis.

Evolutionary conservation analysis showed that the two novel mutations are highly conserved, while the secondary structure and solvent accessibility of wild and mutant types had no significant differences. The predicted protein structure of DAB1 exhibits a different structure. However, the protein-protein interaction network did not show the interaction relationship between DAB1 and NOTCH3, but revealed other proteins that interact with them separately, providing other perspectives for further research. Rare diseases mostly exist individually, and there are fewer cases with both phenotypes, therefore, NGS is a suitable tool for disease diagnosis.

Rare diseases like SCA37 or CADASIL are not rare, either SCA37 or CADASIL has been reported in relevant databases. According to our understanding, this is the first report with two disease phenotypes at the same time, and there are no similar cases in the literature and databases at present. In general in hospitals like ours, in addition to routine diagnosis, it is necessary to use modern technology to diagnose difficult diseases to avoid misdiagnosis or missed diagnosis. In this study, combining molecular biology and conventional imaging methods provided a useful reference for disease diagnosis.

## Data Availability

The datasets presented in this study can be found in online repositories. The names of the repository/repositories and accession number(s) can be found below: NCBI SRR13860596.
